# Original association of ion transporters mediates the ECM-induced breast cancer cell survival: Kv10.1-Orai1-SPCA2 partnership

**DOI:** 10.1038/s41598-018-37602-7

**Published:** 2019-02-04

**Authors:** Marta Peretti, Mehdi Badaoui, Alban Girault, Laurence Van Gulick, Marie-Pierre Mabille, Riad Tebbakha, Henri Sevestre, Hamid Morjani, Halima Ouadid-Ahidouch

**Affiliations:** 1 Laboratory of Cellular and Molecular Physiology, EA4667, University of Picardie Jules Verne, Amiens, France; 2 BioSpecT EA7506, Faculty of Pharmacy, Reims University, Reims, France; 3 Service d’Anatomie et Cytologie Pathologiques and Tumor Bank of Picardie, CHU d’Amiens, Université de Picardie Jules Verne, F-80000 Amiens, France

## Abstract

In the last years it has been shown that many components of tumor microenvironment (TM) can induce cell signaling that permit to breast cancer cells (BC) to maintain their aggressiveness. Ion channels have a role in mediating TM signal; recently we have demonstrated a functional collaboration between Kv10.1 and Orai1 channels in mediating the pro-survival effect of collagen 1 on BC cells. Here we show how SPCA2 (Secretory Pathway Ca^2+^ ATPase) has a role in this process and is able to support survival and proliferation induced by collagen 1. By participating to an auto-sustaining loop, SPCA2 enhances membrane expression of Kv10.1 and Orai1; the activity of every component of this trio is necessary to mediate a store independent calcium entry (SICE). This SICE is fundamental to maintain both the activation of the pro-survival pathway and the membrane localization and consequently the activity of the two channels. Moreover, the three proteins and the collagen receptor DDR1 are overexpressed only in aggressive tumors tissues. In this work, we propose a novel association between SPCA2, Kv10.1 and Orai1 involved in mediating transduction signals from TM to the BC cells that can be potentially exploited in the search of novel therapeutic targets specific to tumor tissues.

## Introduction

Ion channels are membrane proteins that allow the passage of ions between the two sides of the cell plasma membrane. They have fundamental roles in physiological processes and in the last two decades their pathological role in sustaining tumors progression has been underlined. It is now clear that a deregulation of the activity and/or the expression of these channels is able to promote the development of different cancers^1–3^.

Although several studies have demonstrated the role of K^+^ and Ca^2+^ channels in cell proliferation, migration and invasion of different cancers including breast cancer (BC)^4,5^, few studies focused the attention on their specific functional coupling in tumor cells^6–9^. Notably, in breast cancer cells type 3 IP3R (IP3R3) co-localizes and interacts both at molecular and functional levels with BKCa channels^10^ and TRPC1 channels have been shown to control the Ca^2+^ entry mediated by KCa3.1 activation and promote cell proliferation^11^.

Kv10.1 (hEag1) is a voltage activated potassium channel, member of the EAG family, with oncogenic properties and largely expressed in different cancers^4,12^. It was shown to be overexpressed in breast cancer^13^. This channel has been involved in the cell cycle regulation of MCF-7 BC cells^14^. In high invasive BC cells Kv10.1 modulates cell migration in regulating calcium entry through Orai1 channel^15^. In addition, we have recently demonstrated another new functional coupling between Kv10.1 and Orai1, mediating the communication of the cells with the tumor microenvironment in BC^16^. We showed that, in MCF-7 breast cancer cells, collagen 1 is able to induce an anti-apoptotic effect and to promote cells proliferation in serum starved condition. Collagen 1 elicits an increase of Kv10.1 activation that enhances basal Ca^2+^ influx through Orai1, triggering ERK1/2 activation and promoting cell survival.

Orai1 is a calcium channel mainly known for its involvement in Store Operated Calcium entry (SOCE); this role has been shown to be able to sustain BC cells migration^15,17^. Recently it has been underlined a new store-independent (SICE) activation of Orai1^18–20^. In breast cancer cells, Feng and colleagues have demonstrated that SPCA2 (Secretory Pathway Ca^2+^-ATPase 2) is able to interact with and activate Orai1, triggering a calcium entry that does not depend on Stim1 and intracellular calcium stores’ depletion and sustaining cells proliferation. Moreover, the regulation of Orai1 by SPCA2 is not associated with the Ca^2+^ pump activity of SPCA2^18^.

Since it has been shown that Kv10.1 and Orai1 are activated in the response of BC cells to collagen 1^16^, we hypothesized a role for SPCA2 also in this process. We hypothesized that SPCA2 could be able to regulate not only Orai1 activity but also Kv10.1 membrane fractions and to have a role in the interaction between these two actors in BC cells exposed to collagen 1 treatment and in cells survival.

After showing the overexpression of Kv10.1, Orai1 and SPCA2 in similar area of breast cancer tissue, we here demonstrate that SPCA2 has a role in the collagen 1 induced survival of BC cells and that this happens through the regulation of the Kv10.1-Orai1 complex. Moreover, the increased calcium influx observed after collagen 1 treatment is a SICE and is regulated by all the three actors. Specifically, SPCA2 is able to regulate the membrane expression other than the activity of the two channels; this regulation is also calcium dependent. Finally, we show that SPCA2 has a role in regulating Golgi trafficking of Kv10.1.

Our data show for the first time the involvement of such complex, composed by ion transporters, in BC cells as a process induced by tumor microenvironment (TM) signaling.

## Results

### SPCA2, Kv10.1, Orai1 and DDR1 are highly expressed in breast cancer tissues

**Figure 1:**
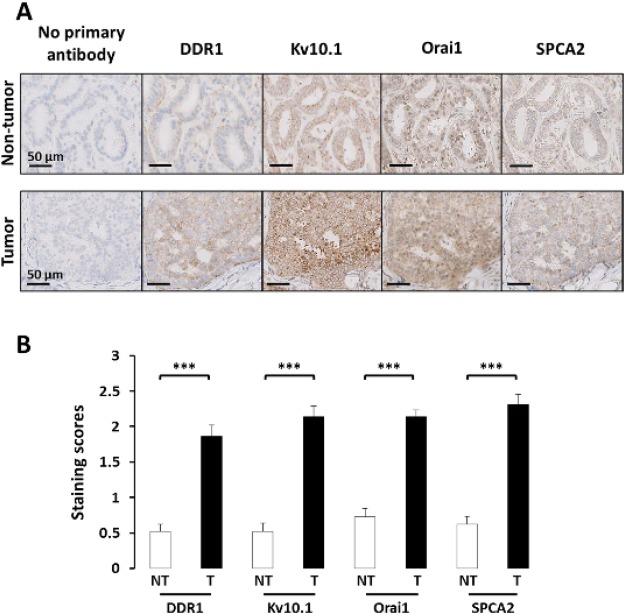
SPCA2, Kv10.1, Orai1 and DDR1 tissue expression. DDR1, Kv10.1, Orai1 and SPCA2 expression in breast cancer tissues. **(A)** Representative immunohistochemical staining of DDR1, Kv10.1, Orai1 and SPCA2 in serial sections of breast tissue. 40x magnifications of tumor (bottom line) is compared to matched normal (up line) tissues. Immunohistochemistry shows the expression of the different proteins in the same area of the sections; staining is higher in tumor areas. **(B)** Comparison of staining scores for DDR1, Kv10.1, Orai1 and SPCA2 in non-tumor vs tumor areas (n = 29; ***p < 0,001, Student T test for paired samples).

We recently demonstrated that Kv10.1 and Orai1 are involved in the regulation of collagen-induced survival of the BC cell line MCF-7. In addition, we observed that this process was closely related to the expression of the collagen-specific receptor DDR1^16^. Indeed, it was reported that collagen is a crucial component of the TM of BC specifically in the promotion of tumor initiation and progression^21^. In our study, collagen 1 was used to mimic the TM of BC cells. We thus decided to perform immunohistochemistry experiments to investigate the expression of the partners in 29 BC samples. Using serial sections, we observed that all of the specimens show staining for all the three partners previously described in cancerous areas (Kv10.1, Orai1 and DDR1; Fig. 1). The intensities of staining are high in cancerous compared to control tissues (Fig. 1B). Moreover, we also analyzed the expression of SPCA2 in consecutive serial sections and we observed its overexpression in the same BC samples compared to healthy ones; this expression was detected in the same areas of consecutive slices as Kv10.1, Orai1 and DDR1.

### SPCA2, as Kv10.1 and Orai1, participates to collagen 1 mediated survival in BC cells

**Figure 2:**
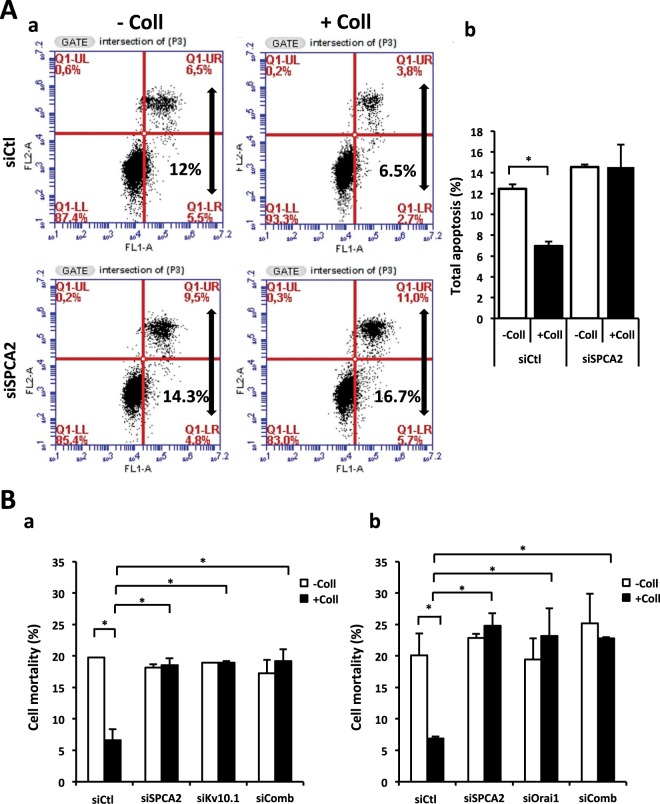
SPCA2 silencing counteracts collagen 1 resistance to apoptosis. **(A)** Effect of SPCA2 silencing on the apoptotic rate of MCF-7 cells. Cells were starved for 48 h and the apoptosis assay was carried out by annexin V/PI staining. (a) Dot blots of a representative experiment and (b) mean ± SEM of total apoptotic cells, N = 3, *p < 0.05, ANOVA followed by Holm-Sidak *post hoc* tests. **(B)** Effect of SPCA2, Kv10.1 and SPCA2 + Kv10.1 silencing (a) or SPCA2, Orai1 and SPCA2 + Orai1 silencing (b) on MCF-7 cell mortality. Cells were starved for 48 h and the mortality was measured by Trypan Blue assay; values are reported as mean ± SEM, N = 3, *p < 0.05, ANOVA followed by Holm-Sidak *post hoc* tests.

Since it was known that in BC cells SPCA2 is able to regulate Orai1 activity, we wanted to investigate whether SPCA2 has a role even in the collagen 1 induced survival mediated by Orai1 and Kv10.1. We studied apoptosis rate after 48 hours of starvation in cells seeded or not on collagen 1. Figure 2A showed that the apoptosis resistance acquired after collagen treatment is lost when SPCA2 is silenced in MCF-7 cells (apoptotic rate (%) siCtl −Coll 12.45 ± 0.45, +Coll 6.95 ± 0.45; siSPCA2 −Coll 14.55 ± 0.25, +Coll 14.55 ± 2.15) as it was demonstrated for Kv10.1 and Orai1. In addition, we analyzed cells mortality in the same conditions with a Trypan Blue assay and we observed the same percentage of loss of collagen 1 pro-survival effect in siSPCA2 MCF-7 cells (Fig. 2B). Moreover, we observed the same effect when we silenced at the same time SPCA2 and Kv10.1 (Fig. 2Ba, Tables 1 and 2 for values) or SPCA2 and Orai1 (Fig. 2Bb) suggesting that all the three proteins participate in the same collagen 1-induced pathway. Moreover, SPCA2 expression seemed not affected by collagen (Supplementary Fig. 1Aa,b), in contrast Orai1 and Kv10.1 expression is increased in the same condition^16^. Supplementary Fig. 1B,C showed the efficiency of transfection with siRNA against Kv10.1, Orai1 and SPCA2. Furthermore, to confirm the role of SPCA2 in collagen 1 mediated survival, we showed that in siSPCA2 cells there is no more pERK1/2 activation, typical of collagen treated BC cells (Supplementary Fig. 1D).

**Table 1:** SPCA2, Kv10.1 and SPCA2 + Kv10.1 silencing inhibit the collagen 1-induced cell survival.

Cell mortality (%)	siCtl	siSPCA2	siKv10.1	siSPCA2 siKv10.1
−Coll	19.76 ± 0.007	18.18 ± 0.5	18.91 ± 0.004	17.27 ± 2.1
+Coll	6.62 ± 1.74	18.54 ± 1.1	18.93 ± 0.26	19.2 ± 1.82

**Table 2:** SPCA2, Orai1 and SPCA2 + Orai1 silencing inhibit the collagen 1-induced cell survival.

Cell mortality (%)	siCtl	siSPCA2	siOrai1	siSPCA2 siOrai1
−Coll	20.11 ± 3.46	22.88 ± 0.60	19.44 ± 3.36	25.20 ± 4.67
+Coll	6.87 ± 0.31	24.81 ± 2	23.18 ± 4.34	22.79 ± 0.19

### Kv10.1, SPCA2 and Orai1 regulate a basal calcium entry in MCF-7 cells in response of collagen 1 coating

In 2010, the work of Feng and collaborators allowed to understand the mechanism of basal activation of Orai1 channels through its coupling with SPCA2^18^. In addition, we have demonstrated recently that collagen 1 increases the calcium entry that is regulated mainly by the cooperation of both channels Kv10.1 and Orai1^16^. We then examined the basal calcium entry in MCF-7 cells and the possible involvement of the SPCA2 protein in the collagen-dependent calcium entry 48 h post-starvation. Moreover, we evaluated whether the calcium influx activated in response to collagen 1 treatment was independent from Ca^2+^ stocks’ depletion and its regulation by the three proteins.

**Figure 3:**
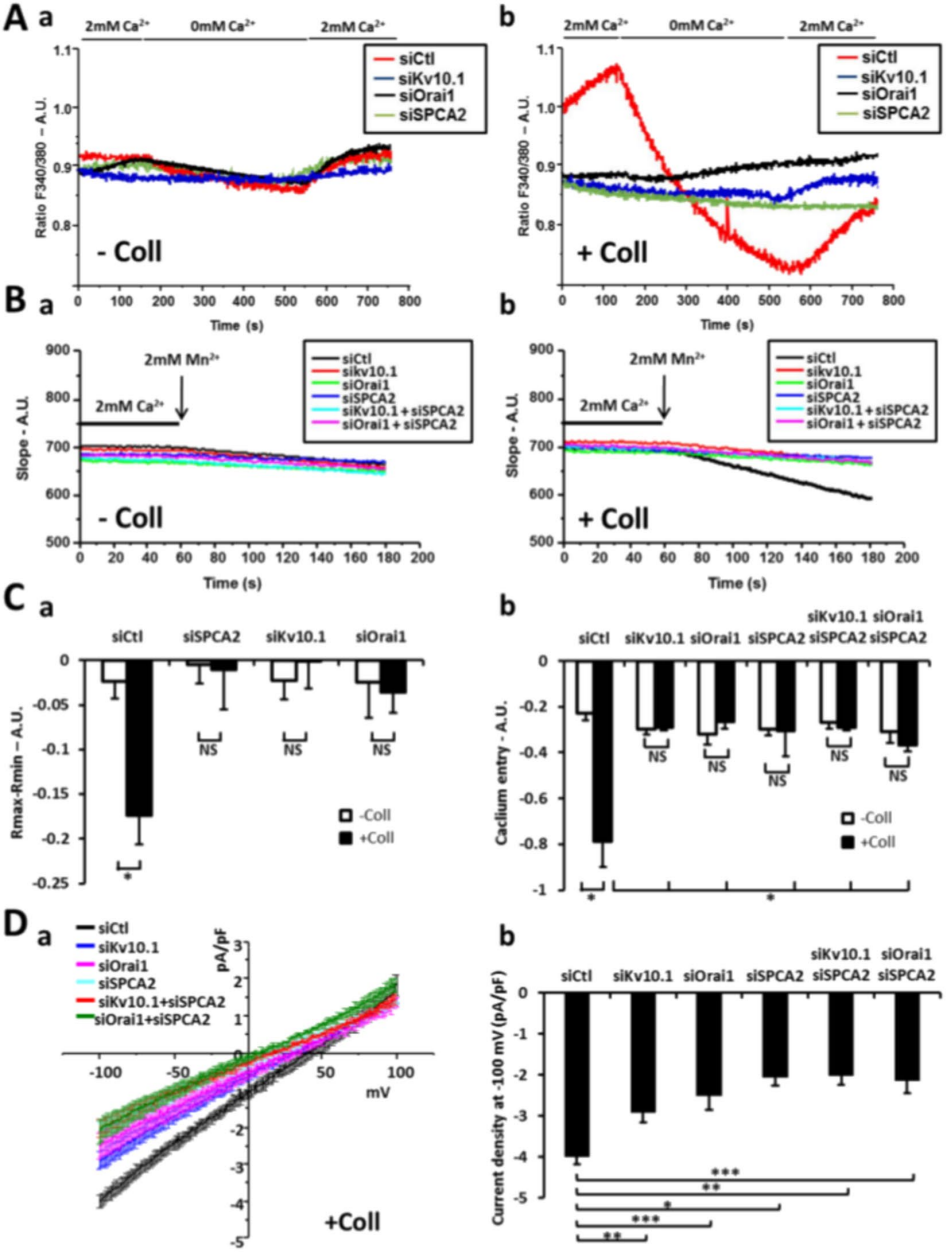
Collagen 1 induced basal calcium entry rely on Orai1, Kv10.1 and SPCA2 activity. **(A)** Representatives traces of basal calcium entry imaging of MCF-7 cells after 48 hours of starvation treated (b) or not (a) with collagen 1. **(B)** Representatives traces of Manganese quench imaging of cells treated (b) or not (a) with collagen 1. Measurements were performed after cells were starved for 48 h **(C)** (a) Histograms representing the averages ± standard error of basal calcium entry (−Coll: n = 90 siCtl, n = 104 siSPCA2, n = 61 siKv10.1, n = 74 siOrai1; +Coll: n = 118 siCtl, n = 86 siSPCA2, n = 76 siKv10.1, n = 69 siOrai1, N = 3; *p < 0.05, ANOVA followed by Holm-Sidak *post hoc* tests). (b) Histograms representing the averages ± standard error of slope values obtained in Manganese quench experiences. (−Coll: n = 104 siCtl, n = 83 siSPCA2, n = 88 siKv10.1, n = 72 siOrai1, n = 91 siSPCA2-Kv10.1, n = 85 siSPCA2-Orai1; +Coll: n = 96 siCtl, n = 71 siSPCA2, n = 77 siKv10.1, n = 87 siOrai1, n = 94 siSPCA2-Kv10.1, n = 98 siSPCA2-Orai1, N = 3; *p < 0.05, ANOVA followed by Holm-Sidak *post hoc* tests). **(D)** Patch clamp recordings of basal calcium currents. (a) Whole cell currents recorded in MCF-7 cells after 48 hours of starvation treated with collagen I. Patch-clamp measurements were performed with specific bath solution to record basal calcium currents. 250 msec ramps from −100 mV to +100 mV from a holding potential of −40 mV were applied. Values are reported as mean ± SEM. (b) Histograms representing the averages ± standard error of currents values at −100 mV. siKV10.1 (n = 6); siCtl and siSPCA2-Kv10.1 (n = 5); siOrai1 and siSPCA2 (n = 4); siSPCA2-Orai1 (n = 3); *p < 0.05, **p < 0.01, ***p < 0.001, ANOVA followed by Tukey *post hoc* tests.

First, we observed that the fluorescence ratio, in the presence of the normal extracellular medium (2 mM Ca^2+^), was significantly higher in siCtl MCF-7 cells seeded on collagen 1 compared to other conditions (Fig. 3Ab,Ca, −Coll siCtl 0.85 ± 0.01, +Coll siCtl 1.06 ± 0.02). Moreover, siCtl MCF-7 treated with collagen 1 showed a very significant decrease in the fluorescence ratio during the perfusion of the 0 mM Ca^2+^ extracellular medium in contrast to cells not treated with collagen seeded on plastic indicating a greater basal calcium entry induced in the presence of collagen 1 (Fig. 3Ab); this effect was abolished when SPCA2, Kv10.1 or Orai1 were silenced (Fig. 3A,Ca, siCtl −0.17 ± 0.03, siSPCA2−0.01 ± 0.04, siKv10.1−0.001 ± 0.03, siOrai1−0.03 ± 0.02). We obtained the same profiles evaluating Ca^2+^ entry with Mn^2+^ quench protocols (Fig. 3B,Cb). Moreover, with this protocol, we observed that there was no additive effect after the double silencing of SPCA2-Kv10.1 or SPCA2-Orai1 (Fig. 3B,Cb, +Coll siCtl −0.78 ± 0.11, siSPCA2−0.3 ± 0.11, siKv10.1−0.28 ± 0.01, siOrai1−0.26 ± 0.02, siSPCA2-Kv10.1−0.28 ± 0.01, siSPCA2-Orai1−0.36 ± 0.03). The recording of basal Ca^2+^ currents with patch clamp experiments confirm the inhibition of the currents stimulated by collagen 1 treatment in siSPCA2, siKV10.1, siOrai1 MCF-7 cells and also in siSPCA2-Kv10.1 and siSPCA2-Orai1 cells (Fig. 3Da,b, average current density values at −100 mV: siCtl −3.99 ± 0.24 pA/pF, siOrai1−2.45 ± 0.45 pA/pF, siKv10.1−2.06 ± 0.28 pA/pF, siSPCA2−2.88 ± 0.33 pA/pF, siSPCA2-Orai1−2.13 ± 0.32 pA/pF, siSPCA2-Kv10.1−1.87 ± 0.23 pA/pF). Finally, we verified the effect of collagen 1 on SOCE using specific protocols for Ca^2+^ imaging (Supplementary Fig. 2A), and patch clamp (Supplementary Fig. 2B). From these experiments, neither collagen nor siKv10.1, Orai1 and SPCA2 affected SOCE. Moreover, siStim1 transfected cells were used as a control of SOC entry inhibition (Supplementary Fig. 2A). All these results suggest that collagen 1 induced a basal calcium entry independently of the reticular calcium release through the participation of the three actors: Kv10.1, Orai1 and SPCA2.

### SPCA2, in response to collagen 1, co-localizes and interacts with Kv10.1 and Orai1

**Figure 4:**
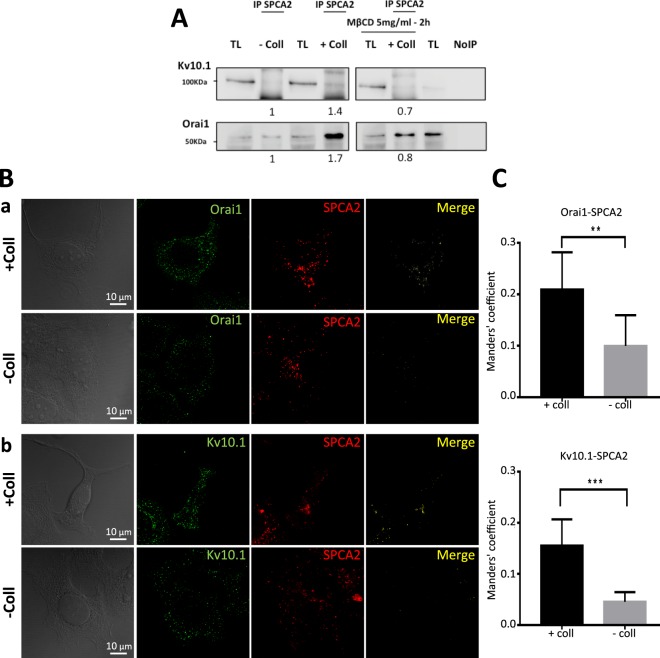
SPCA2 co-localizes and interacts with Kv10.1 and Orai1. **(A)** Effect of collagen 1 alone and with MβCD on SPCA2 interaction with Orai1 or Kv10.1. Representative western blot of Orai1 and Kv10.1 expression after immunoprecipitation with an anti-SPCA2 antibody. Results were normalized as a percentage of the untreated control condition (−Coll). Values are reported as means of all the experiments, N = 3. **(B)** Staining of (a) Orai1 (green) and SPCA2 (red) and their merge or (b) Kv10.1 (green) and SPCA2 (red) and their merge to visualize co-localization in MCF-7 cells treated or non-treated with collagen 1 after 48 h of starvation. **(C)** Histograms showing the means of the Manders Overlap Coefficient of co-localization of Orai1-SPCA2 and Kv10.1-SPCA2 in cells treated or not with collagen 1. (N = 2, n = 35; *p < 0.05, **p < 0.01, Two samples *t*-test)

To verify whether, in addition to its participation to the same calcium pathway, SPCA2 was also co-localized with the two channels, we first carried out immunoprecipitation experiments with an anti-SPCA2 antibody. As shown in Fig. 4A, MCF-7 cells, when seeded on collagen 1 coating, show an increased physical interaction of SPCA2 with both Kv10.1 and Orai1 after 48 hours of starvation. Furthermore, treatment of cells with MβCD, which leads to cholesterol depletion and disruption of lipid raft microdomains, decreased collagen-induced interaction between SPCA2 and Kv10.1 or Orai1. This suggests that the interaction takes place at the level of lipid rafts (Fig. 4A). In addition to this, Kv10.1 immunoprecipitation showed that the interaction between Kv10.1 and Orai1 is decreased when SPCA2 is silenced (Supplementary Fig. 3). We then confirmed these data by using immunofluorescence-staining experiments (Fig. 4B). This increase in the co-localization was significant when analyzed by the Manders Overlap Coefficient (Fig. 4C; Orai1 and SPCA coefficient values: +Coll 0.209 ± 0.072 vs −Coll 0.099 ± 0.059 and Kv10.1 and SPCA coefficient values: +Coll 0.155 ± 0.051 vs −Coll 0.046 ± 0.019). High magnification images presented in Supplementary Figures 5 and 6 precise that Orai1 and SPCA2 co-localized closely to the plasma membrane and Kv10.1/SPCA2 complexes are detected preferentially at the intracellular level.

### SPCA2 regulates the membrane enrichment of Kv10.1 and Orai1 and Kv10.1 activity

**Figure 5:**
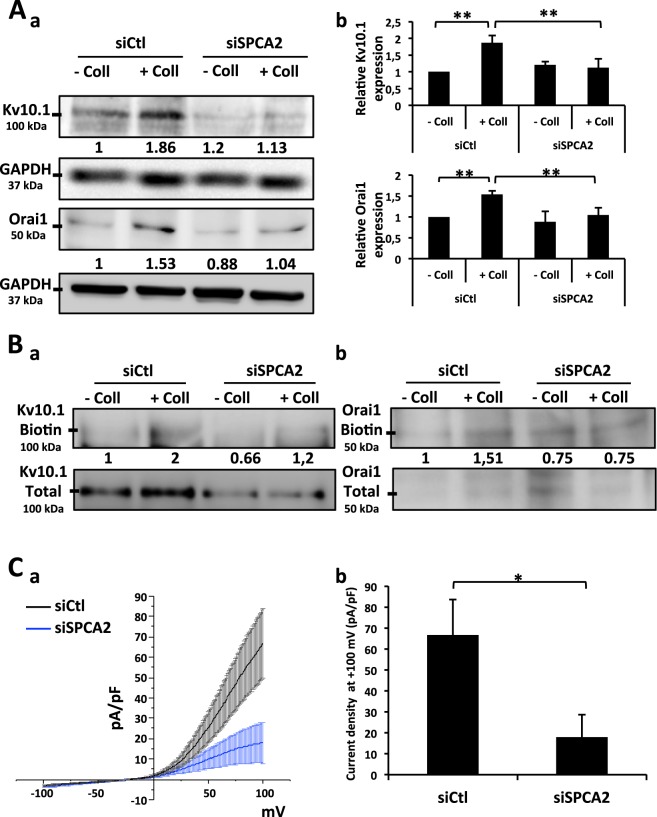
SPCA2 regulates Kv10.1 and Orai1 total and membrane expression. **(A)** Representative western blots (a) of Kv10.1 and Orai1 proteins levels in siCtl and siSPCA2 MCF-7 cells after 48 h of starvation treated or not with collagen 1. Results were normalized as a percentage of the untreated control condition (−Coll). Values are reported as means of all the experiments. (b) Histograms representing the averages ± standard error of results obtained by western blots. N = 5, **p < 0.01**;** ANOVA followed by Holm-Sidak *post hoc* tests. **(B)** Effect of SPCA2 silencing on Kv10.1 and Orai1 membrane expression. Representative western blots showing membrane fraction enrichment of Kv10.1 (a) and Orai1 (b) proteins in siCtl and siSPCA2 MCF-7 cells after 48 h of starvation. The membrane fractions were obtained through a biotinylation protocol. Results were normalized as a percentage of the siCtl collagen 1 treated condition. Values are reported as mean of all the experiments. N = 3. **(C)** Whole cell currents (a) recorded in siCtl and siSPCA2 MCF-7 cells after 48 h of starvation treated with collagen 1. 500 msec voltage ramps from −100 to +100 mV from a holding potential of −40 mV were applied to record Kv10.1 channel activity. Values are reported as mean ± standard error. (b) Histograms representing the averages ± standard error of currents values at +100 mV. siCtl (n = 10), siOrai1 (n = 5); *p < 0.05; Two-samples *t*-test.

We have previously shown that collagen 1 induces Kv10.1 and Orai1 overexpression^16^. Since now we found that the inhibition of SPCA2 expression completely reduced the collagen 1 effect, we first studied the effect of SPCA2 silencing on the collagen 1-induced overexpression of Kv10.1 and Orai1. We performed western blot experiments to monitor the protein levels of Kv10.1 and Orai1. As shown in Fig. 5A in siSPCA2 cells, the expression of both the proteins is reduced.

It is known that SPCA2 interacts with Orai1 and regulates its trafficking^19^. Then, by using biotinylation assays we demonstrated that collagen 1 induces enrichment in membrane fractions of both Kv10.1 and Orai1. This increase is counteracted by the silencing of SPCA2 (Fig. 5B).

We previously demonstrated that in our cell culture conditions, the most important contribution of the potassium conductance was due to Kv10.1 channel in MCF-7 cells^16^. In agreement with the impact of SPCA2 on the expression of Kv10.1 and Orai1 and the importance of Kv10.1 activity in MCF-7 cells, we observed that when SPCA2 is silenced there is a decrease in Kv10.1 channel activity represented by a low current density in patch clamp recordings (Fig. 5C, average current density values at +100 mV siCtl 66.65 ± 17 pA/pF, siSPCA2 26.55 ± 4.76 pA/pF).

### Ca^2+^ levels and membrane protein enrichment participate in an auto-sustaining loop

**Figure 6:**
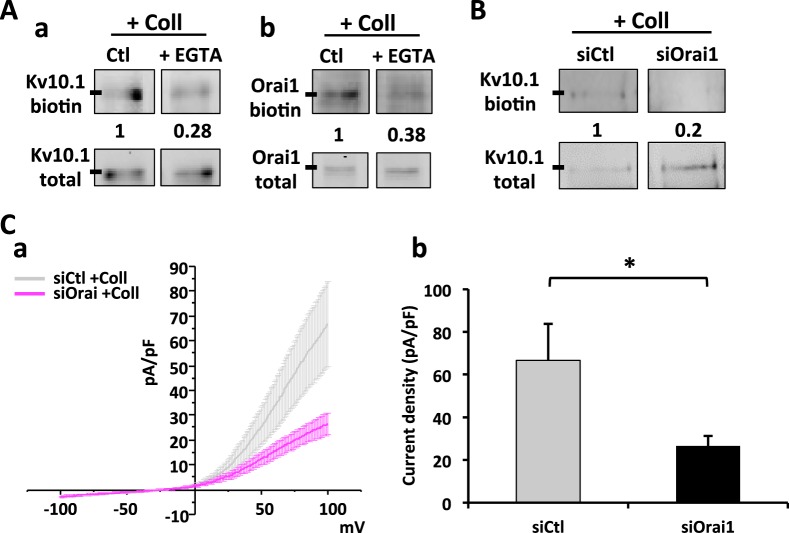
Orai1 membrane fraction depends on calcium levels; Kv10.1 membrane fraction depends on calcium levels and Orai1. **(A)** Representative western blots showing membrane fraction of Kv10.1 (a) and Orai1 (b) proteins in MCF-7 cells after 48 h of starvation treated or not with EGTA 1.6 mM. The membrane extracts were obtained through a biotinylation protocol. Results were normalized as a percentage of control condition. Values are reported as mean of all the experiments. N = 3. **(B)** Representative western blot showing membrane fraction enrichment of Kv10.1 in siCtl and siOrai1 MCF-7 cells after 48 h of starvation. The membrane extracts were obtained through a biotinylation protocol. Results were normalized as a percentage of siCtl condition. Values are reported as mean of all the experiments. N = 3. **(C)** Whole cell currents (a) recorded in siOrai1 MCF-7 cells after 48 h of starvation treated with collagen 1. 500 msec voltage ramps from −100 to +100 mV from a holding potential of -40 mV were applied to record Kv10.1 channel activity. Values are reported as mean ± standard error. (b) Histograms representing the averages ± standard error of currents values at +100 mV. The siCtl values reported in gray came from the same recording as in Fig. 5C. siOrai1 (n = 8); *p < 0.05; Two-samples *t*-test.

We investigated whether the collagen 1-induced increase in membrane expression of Kv10.1 and Orai1 was sustained by the calcium entry. To monitor this, we checked if the membrane fractions of the channels were modulated by changes in calcium levels. In Fig. 6A we show that when cells are treated during the starvation period with EGTA at a concentration of 1.6 mM (predicted free external [Ca^2+^] = 0.2 mM), the collagen 1-induced enrichment in membrane fraction of both the channels was decreased. Since all the data point to a tendency to a mutual regulation of the three components, we analyzed the effect of Orai1 silencing on Kv10.1. Figure 6B shows that the expression of Kv10.1 in membrane fractions of MCF-7 cells seeded on collagen 1 is decreased when Orai1 expression is impaired by siRNA. In addition, patch clamp measurements of potassium current densities in siOrai1 MCF-7 cells are significantly lower than those observed in siCtl cells (Fig. 6C, average current density values at +100 mV siOrai1 17.95 ± 10.72 pA/pF).

### Kv10.1 but not Orai1 trafficking through the Golgi apparatus is regulated by collagen 1 and SPCA2

**Figure 7:**
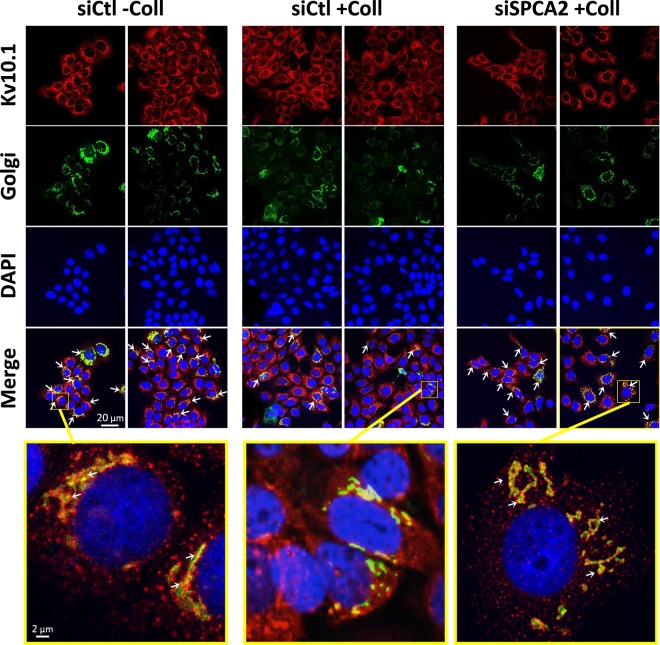
Collagen 1 stimulates Kv10.1 departure from Golgi through a SPCA2-dependent pathway. Confocal microscope images of MCF-7 cells infected with cell light Golgi-GFP vector at the beginning of the starvation and then immunostained with an anti-Kv10.1 antibody. The conditions analyzed were siCtl treated or not with collagen 1 and siSPCA2 treated with collagen 1. The white arrows indicate the co-localization between Kv10.1 and Golgi.

Since SPCA2 is a Golgi resident Ca^2+^ ATPase, we decided to verify if the Golgi trafficking of the two channels to the membrane was influenced by collagen 1 and/or regulated by SPCA2. In order to achieve this, we performed immunofluorescence analysis of Kv10.1 and Orai1 in MCF-7 cells treated with the cell light Golgi-GFP at the moment of the starvation. In Fig. 7 we show staining for Kv10.1 (red), the Golgi (green) and the cells nuclei with DAPI (blue). Orai1 does not show co-localization with the Golgi but only a membrane localization that is increased when cells were seeded on collagen 1 (Supplementary Fig. 4). Regarding Kv10.1, we observed a marked co-localization of the channel with Golgi apparatus in absence of collagen 1. This co-localization is decreased when the cells are seeded on collagen 1. In this condition, the silencing of SPCA2 leads to a Kv10.1 localization pattern more similar to cells in the absence of collagen (closely related to the Golgi staining) (Fig. 7). In addition, we observed a similar modification of the localization of Kv10.1 in human tissue. Indeed, normal tissue has a reduced expression and a more colocalized pattern of Kv10.1 with trans-Golgi network compared to the tumor tissue (Supplementary Fig. 7).

## Discussion

Mammary gland represents an organ where the stroma plays a very important role in its development. Although BC is the second most commonly diagnosed cancer in the world only in recent years studies on the role of the TM in development of this cancer have been published. Indeed, it has been shown that many TM components can regulate tumor progression^22^.

In this work we deeply investigate the cellular mechanisms mediating the pro-survival effect of collagen 1, the most abundant matrix protein in breast^23^, which is correlated with a higher risk of BC development, especially in relation to its density^24^. We demonstrate for the first time a role for SPCA2 in this process. This happens through SPCA2 regulation of the localization and the activity of Kv10.1 and Orai1, mediating a store-independent calcium influx able to sustain channels membrane localization and activity and to promote cells survival.

Here we show for the first time at tissue level, the presence of all these three proteins in the same areas of aggressive breast tumors. A fourth protein also overexpressed in the very same areas is DDR1, the collagen 1 receptor who mediates its pro-survival effect in BC cells (Fig. 1). In non-tumor sections these proteins are either not expressed (DDR1 and SPCA2) or faintly expressed (Orai1 and Kv10.1), suggesting the importance of their simultaneous expression to sustain the aggressiveness and the resistance of the tumor.

At a cellular level, we demonstrate here that SPCA2 acts in the pro-survival pathway activated by collagen 1 and that the pathway is common with Kv10.1 and Orai1 (Fig. 2, Supplementary Fig. 1). In this collagen mediated survival process, the three proteins act collectively to activate a basal calcium influx independently from SOC pathways. In fact when at least one of the three elements is silenced, the basal calcium influx is suppressed (Fig. 3, Supplementary Fig. 2). These results are in agreement with previous works that have associated the Orai1 channel with SOCE in ER^−^ BC cells, and with SICE in ER^+^ BC cells (MCF-7 and T-47D)^18,25^. SPCA2 has already been reported to be able to interact with and regulate Orai1 activating SICE^18,19^; we now propose this mechanism as a part of the BC cells response to TM stimulation and we add Kv10.1 as the third actor in this machinery. Both Kv10.1 and Orai1 co-localize with SPCA2 and physically interact with it. We demonstrated here that these interactions could take place at plasma membrane level or very close to it with some interactions of the Golgi apparatus network (Figs 4, 7 and Supplementary Figs 5 and 6). The different but very close situation of the partners in the plasma membrane and in the vesicle compartment of Golgi apparatus could also explain the low value of Manders Overlap Coefficient. In addition, we observed modifications of the localization of Kv10.1 in normal and cancerous human tissue (Supplementary Fig. 7). Remarkably, this interaction seems dependent on the stability of lipid rafts (Fig. 4).

While SPCA2 expression do not change in response to collagen (Supplementary Fig. 1), here we observed that its presence is fundamental for the increase of Kv10.1 and Orai1 expression when cells are seeded on collagen 1. Very interestingly, not only the expression of Kv10.1 and Orai1 are increased in response to collagen 1 but especially their membrane trafficking; moreover, the membrane localization of both the channels relies on the presence of SPCA2. Furthermore, the silencing of SPCA2 when cells are seeded on collagen 1 not only affects the membrane localization of both channels but also their activity (Fig. 5). The impact of TM on the localization of ion channels in cancer is poorly explored. Indeed, until now only two studies described this process; the first demonstrated that a pro-domain of MMP-23 is able to induce an intra-cytoplasmic localization of Kv1.3^26^, whereas Tenascin-C, a glycoprotein expressed in the TM of various tissues, is able to increase the membrane localization of Nav sodium channels in neurons^27^. Our study demonstrates that, in response to collagen 1 stimuli in BC highly proliferating cells, the SPCA2-Kv10.1-Orai1 trio is co-localized at a membrane level. Triggered by the interaction with SPCA2, the membrane fractions and the activity of the two channels are boosted, sustaining a store-independent calcium entry. The K^+^ efflux mediated by Kv10.1 is probably needed as shown in MDA-MB231 cells to shift the membrane potential towards negative values therefore increasing the driving-force for Ca^2+^ ^15^. In this case, hampering the activity of one of the three will lead to the loss of the collagen 1-induced pro-survival effect.

We have previously shown that in the collagen 1-induced pro-survival pathway there is a loop between the two channels expression and ERK phosphorylation, specifically connected to an increase in calcium entry^16^. Here, we reveal another part of the loop involving Ca^2+^ levels. As the matter of fact the membrane fraction of both Kv10.1 and Orai1 is reduced when external Ca^2+^, and consequently Ca^2+^ influx, is reduced. Thus, the calcium influx mediated by Orai1 is necessary also to maintain the membrane localization of both Orai1 and Kv10.1 (Fig. 6). A Ca^2+^ regulation of ion channels membrane insertion has already been shown by Cheng and colleagues; Ca^2+^ entry via Orai1/STIM1-CRAC channel triggers plasma membrane insertion of TRPC1. After its membrane insertion and the interaction with STIM1, Ca^2+^ entry mediated by TRPC1 is the primary regulator of KCa channel^28^.

According to the all provided data, we can hypothesize that in our system there should be a regulation by SPCA2 and Ca^2+^ levels on the protein-trafficking pathways to the plasma membrane. Calcium has also a role in the regulation of some intracellular membrane fusion processes and consequently in protein trafficking. As an example, an important role has been reported for Ca^2+^in the trafficking from endoplasmic reticulum to the Golgi or during early endosome fusion^29^. Regarding the intra-Golgi trafficking there is a specific regulation of Ca^2+^ concentration. Indeed, it is required in a narrow concentration range for this process^30^.

It is well known that Orai1 regulation by SPCA2 is independent from SPCA2 Golgi activity^18,20^. In our work, we confirm that there is no localization of Orai1 in the Golgi apparatus either in cells seeded or not on collagen 1 (Supplementary Fig. 4). Thus, the Ca^2+^ regulation of Orai1 trafficking passes through an unknown Golgi-independent pathway. To address this question requires a full separate study.

Kv10.1 is one of the proteins that are processed in the Golgi for post-translational modification, notably glycosylation. This modification has an impact on both the protein trafficking and its functional properties as a channel^31^. Here we observed that Kv10.1 is less retained in the Golgi when cells are seeded on collagen 1 and in the presence of SPCA2 (Fig. 7). Thus, Kv10.1 trafficking relies on Golgi complex; the effect of SPCA2 on this trafficking could be either direct, connected to its Golgi Ca^2+^-ATPase function^32^, or indirectly associated to the effect on intracellular Ca^2+^ concentration. Regarding the mechanisms involved in the Golgi sorting of Kv10.1, we can speculate on two different hypothesis that can be confirmed with further investigations. The first one involves the effect of Ca^2+^ on membranes fusion at the Golgi level. Another possibility can be related to the role of calcium regulation of glycosylation process. Indeed, it has been demonstrated that the glycosylation of thyroglobulin can be affected by changing Ca^2+^ concentration^33^. Moreover, the yeast Gdt1, a Golgi-localized calcium transporter, has been reported to be required for proper protein glycosylation^34^. In a similar scenario Kv10.1 glycosylation and consequently its sorting could be dependent on Ca^2+^ concentration.

**Figure 8:**
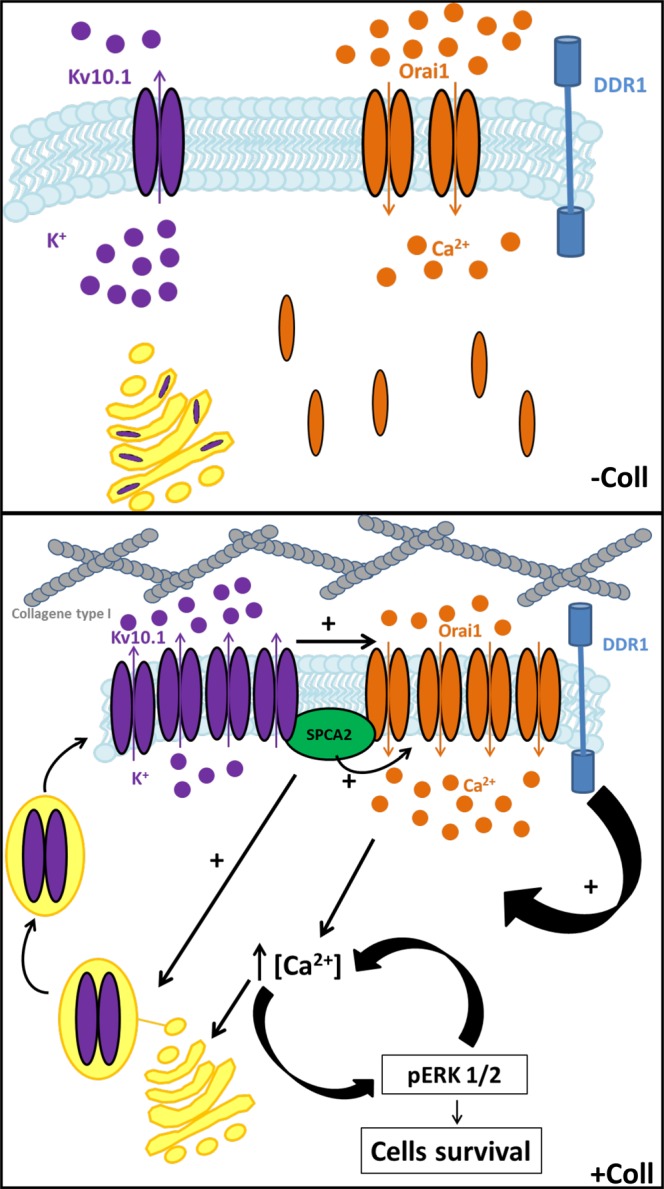
Schematic summary of the proteins and pathways activated by Collagen 1 treatment in BC cells.

In conclusion of our work, we propose a novel association between SPCA2, Kv10.1 and Orai1 involved in a signal transduction pathway mediated by TM in BC cells. The complex, through the membrane insertion and activity of the two channels sustained by the interaction with SPCA2, promotes a basal calcium influx. This influx on one hand promotes cells proliferation and survival and on the other hand auto-sustain the complex activity (Fig. 8). Thus, the complex constituted by these three components has a fundamental role in promoting the aggressiveness of malignant cells. Since we have observed that the three proteins, together with the collagen 1 receptor DDR1, are co-expressed in aggressive tumors tissues, targeting one or more than one of these four actors would represent an attractive therapeutic strategy in BC treatment. Indeed, in this scenario, the alteration of expression, localization or function of one of the components of this new partnership would lead to the disruption of the TM-induced pro-survival pathways specific of BC cells and consequently inhibit the tumor progression.

## Materials and Methods

### Cell culture and collagen 1 extraction and preparation

MCF-7 cells culture was conducted as previously described^16^. Collagen 1 has been extracted and prepared as previously described^16^. All the experiments are conducted on cells after 48 hours of starvation in medium depleted of FBS. Cells are seeded in complete medium in presence or absence of collagen 1 coating (2.5 µg/cm^2^) and after 24 hours the starvation is induced with a switch to serum free-medium.

### Cell transfection and RNA interference

Transfection of cells was performed using nucleofection technology (Amaxa Biosystems, Lonza, Aubergenville, France) according to the protocol previously described^11^. Cells were transiently transfected with siRNA directed against Kv10.1 (Dharmacon Research, Chicago, IL), Orai1 (Dharmacon Research, Chicago, IL), SPCA2 (Kaneka Eurogentec S.A.,Seraing, Belgium) and Stim1 (Santa Cruz Biotechnology, Inc., Heidelberg, Germany), or with scrambled siRNA as a control (siCtl) (siGENOME Non-Targeting siRNA, Dharmacon Research, Chicago, IL), and used 72 h after transfection.

### Cell mortality

The cell mortality was assessed by Trypan blue assay as previously described^16^.

### Western blotting

Proteins were extracted, quantified and separated as previously described^16^. The primary antibodies used were: anti-Kv10.1 (1:200, Santa Cruz Biotechnology, Inc., Heidelberg, Germany), anti-Orai1 (1:200, Sigma Aldrich, Saint-Quentin-Fallavier, France), anti-SPCA2 (1:250, Santa Cruz Biotechnology, Inc.), anti-ERK1/2 (1:500, Cell Signaling Tech., Danvers, USA), anti-p-ERK1/2 Thr202/Tyr204 (1:500, Cell Signaling Tech.). GAPDH (1:1,000, Cell Signaling Tech.) antibody was used for loading control experiments. Detection and quantification were realized as previously described^16^.

### Kv10.1, Orai1 and SPCA2 staining

MCF-7 cells were washed (1 mM MgCl_2_, 100 mM KCl and 20 mM HEPES at 37 °C), then fixed and permeabilized using 4% paraformaldehyde in PBS supplemented with 40 μg/mL digitonin for 10 min. Before staining, cells were washed three times with PBS and non-specific sites were saturated for 45 min with 10% normal goat serum (NGS) at room temperature. The anti-Kv10.1 (Alomone Labs, Jerusalem, Israel) or anti-Orai 1 (ProSci Inc, CA, USA) antibodies were added at dilution of 1:100 for 1 hour and detected in a first step after a 3% NGS saturation with a biotinylated anti-goat secondary antibody (1:50, Jackson ImmunoReasearch Labs, Inc, PA, USA). Then, after a second NGS saturation at 10%, the SPCA2 antibody (Santa Cruz Biotechnology, Dallas, USA) was added at dilution of 1:75 for 1 hour. Finally, in the second step a streptavidin AlexaFluor® 488 conjugate antibody (1:500, Thermo Fisher Scientific, Villebon sur Yvette, France) and a secondary AlexaFluor® 568 conjugate antibody (1:100, Invitrogen, Cergy-pontoise, France) were used for Kv10.1 or Orai1 detection and SPCA2 detection respectively.

### Image processing and analysis

For fluorescence image acquisition, confocal microscope LSM710 and ZEN software (Carl Zeiss MicroImaging, LLC, Oberkochen, Germany) were used. Images were analyzed with ImageJ Software. Manders Overlap Coefficient was analyzed using “JACoP” plugin^35,36^.

### Patch clamp experiments

For electrophysiological analysis, patch clamp analysis were realized as previously described^16^.

### Calcium imaging

Cellular calcium imaging was used to investigate the Store Independent Calcium Entry (SICE), the basal calcium entry and the Store Operated Calcium Entry (SOCE). Briefly, MCF-7 cells were seeded on glass coverslips in 35 mm petri-dishes at a density of 10*10^4^ cells and starved for 48 h. Cells were loaded with Fura-2 AM at 3 μM in extracellular recording solution for 45 min at 37 °C (in mM): NaCl 145, KCl 5, CaCl_2_ 2, MgCl_2_ 2, HEPES 10, glucose 5 and pH adjusted to 7.4 (NaOH). Cells were washed twice and then excited at 340/380 nm with a monochromator (TILL® Photonics, Munich, Germany) or 360 nm (the isosbestic point of Fura-2 AM) with regards to the Manganese quenching experiments. Finally, Fura-2 emission was recorded at 510 nm by a CCD camera coupled to a Zeiss inverted microscope (Carl Zeiss MicroImaging, LLC, Oberkochen, Germany). The SICE was analyzed by following the variation of the ratio F340/380 (which corresponds to the intracellular calcium basal concentration) only by changing the calcium concentrations of the extracellular medium through the perfusion of 2 mM Ca^2+^ to calcium-free extracellular medium (0 mM Ca^2+^). Concerning the SOCE, cells were perfused by thapsigargin at 1 μM in free calcium extracellular medium inducing reticular calcium release, the cells were then perfused by calcium-rich extracellular medium (10 mM Ca^2+^). The Mn^2+^ quenching experiments were performed as previously described^16^.

### Apoptosis analysis

To estimate the percentage of apoptotic cells, we studied cell surface phosphatidylserine exposure, an early marker of apoptotic cell death, by performing a PE Annexin V Apoptosis Detection Kit I staining (BD Biosciences Pharmingen, Le Pont de Claix, France) as described^16^.

### Immunohistochemistry

The study was approved by the “Comité de Protection des Personnes Nord-Ouest II”. Informed consent was obtained from each patient. All procedures were performed in accordance with the Helsinki Declaration of 1975, revised in 1983. Twenty-nine human breast ductal adenocarcinoma specimens were obtained from women having undergone operations at the Amiens hospital. Additionally, normal tissue (used in Supplementary Fig. 7) was obtained with non-opposition of research used consent. Clinicopathological parameters of patients can be found in Table 3. Immunohistochemical studies were performed using the indirect immuno-peroxidase staining technique on the paraffin-embedded material with a Ventana Benchmark Ultra instrument (Ventana Medical Systems, Roche Diagnostics, Meylan, France) and a hematoxylin counterstain as previously described^37^. Briefly, after blocking the endogenous peroxidase by the I-View Inhibitor (Ventana), sections were stained with an anti-Kv10.1 (1/100, Santa Cruz) or anti-Orai1 (1/100, Sigma Aldrich) or anti-SPCA2 (1/100, Santa Cruz) or anti-DDR1 (1/100, Cell Signaling) for 32 min, washed, incubated with ultra View Universal DAB Detection Kit (Ventana) for 8 min. DAB/H_2_O_2_ was used as chromogen and the slides were then examined under optical microscopy. Immunostaining of the tumor tissue was determined by subjective visual scoring of the brown stain. Kv10.1 expression was evaluated by the estimation of staining intensity, which was rated on a scale of 0–3, with 0 = negative; 1 = weak; 2 = moderate; 3 = strong. Micrograph acquisitions were performed by using a Leica slide scanner (SCN 400, Leica Biosystems Germany).

**Table 3:** Clinicopathological parameters of the 29 patients. Abbreviation: ER, Estrogen Receptor.

Total	Median age (range)	Tumor size		Grade			Ki67		ER status		Lymph node metastasis	
		≤2 cm	>2 cm	I	II	III	≤10%	>10%	Neg.	Pos.	Neg.	Pos.
29	61 (30–80)	6	23	3	15	10	9	17	5	22	7	22

### Co-Immunoprecipitation

Co-Immunoprecipitation experiments were performed to investigate the physical interaction between the different proteins. Following the RIPA protein extraction, 1 mg of proteins contained in 300 μl of RIPA were precleared with the magnetic beads A for 2 h at 4 °C (Pure Proteome™, Millipore). Then, the supernatant was incubated overnight with 4 μg of anti-Kv10.1 or anti-SPCA2 (Santa Cruz Biotechnology, Inc., Heidelberg, Germany) at 4 °C, then 50 μl of beads are added for 1 h at 4 °C inducing the precipitation of the protein/antibody complexes. The beads are washed, the complexes are desaturated and the proteins analyzed by Western Blot. The loading control for IP experiment is LT. It represents 50 µg from the same total cell lysate used for the co-IP experiments and it was used as a positive control in order to evaluate if the band in the IP lanes appears at the right molecular weight. To analyze the protein interaction localization, MCF-7 cells were treated by methyl-β-cyclodextrin (MβCD, Sigma Aldrich, Saint-Quentin-Fallavier, France), a disruptive agent for lipid rafts, for 2 h at a concentration of 5 mg/ml.

### Biotinylation assay

To evaluate the differences in the membrane expression of the protein we performed biotinylation assays as following. 5*10^5^ cells are plated on a P60 Petri-dish. Cells are washed with cold PBS and incubated with 2 mg of Sulfo-NHS-SS-biotine (Thermo Fisher Scientific, Rockford, IL) 45 minutes at 4° with shaking. The reaction is stopped by wash with cold PBS + 10 mM glycine. Cells are scraped in RIPA buffer. Ten per cent of the extract is kept for total protein samples and the rest is incubated overnight at 4° with streptavidine agarose beads (Thermo Fisher Scientific, Rockford, IL) pre-washed with RIPA. Beads are washed 4 times with RIPA buffer. Proteins are eluted with 50 µl of Laemmli 2X and heating 60° for 30’. Proteins are separated by denaturing SDS–PAGE, transferred onto nitrocellulose membranes and incubated with specifics antibodies for western blot.

### Immunofluorescence and Golgi staining

To evaluate the localization of our proteins relative to Golgi apparatus, cells were treated with cell light Golgi-GFP (Life Technologies, Eugene, OR; 5 μl/10000 cells) 48 hours before fixation, simultaneously of cells starvation. Cells were fixed with paraformaldehyde 4% for 20 minutes, permeabilized with triton 0.5% and blocked for 30 minutes with BSA 3%. The cells are then incubated overnight with primary antibodies (Kv10.1 1/50, Santa Cruz Biotechnology, Inc., Heidelberg, Germany; Orai1 1/200, Sigma Aldrich, Saint-Quentin-Fallavier, France; SPCA2 1/100, Santa Cruz Biotechnology, Inc., Heidelberg, Germany), washed with PBS, incubated 1 hour with secondary antibodies conjugated with fluorophores (anti-mouse Alexa 546, 1/200, Life Technologies, Eugene, OR; anti-rabbit Alexa 549, 1/200, Thermo Fisher Scientific, Rockford, IL) and counterstained with DAPI to visualize the nuclei. Analyses were performed on a LSM 780 confocal microscope (Carl Zeiss) and analyzed with ZEN 2012 software. For immunofluorescence assay on human tissue, samples were stained with anti-Kv10.1 (1/50, Alomone Labs, Jerusalem, Israel) and TGN46 (1/100, Thermofisher Scientific).

### Statistical analysis

Data are presented as mean ± SEM (standard error of mean), n refers to the number of cells, and N refers to the number of cell line passages. All the experiments were performed in triplicate in at least 3 different cell lines passage number. The mean values of two groups were compared by the Student’s *t*-test or Mann-Whitney rank sum test, using Sigma-Stat 3.0 (Systat Software, Inc.), differences between the values were considered significant when *p* < 0.05. The *p*-values < 0.05, <0.01, and <0.001 are represented as *,**, and ***, respectively. Mean values of more than two groups were tested using one or two-way analysis of variance (ANOVA) followed by Tuckey or Holm-Sidak *post hoc* tests, differences between the values were considered significant when *p* < 0.05. The *p*-values < 0.05 are represented as*.

The datasets generated during and/or analyzed during the current study are available from the corresponding author on reasonable request.

## Supplementary information

Supplementary material
