# Supplementary material for: Original association of ion transporters mediates the ECM-induced breast cancer cell survival: Kv10.1-Orai1-SPCA2 partnership
Source: Sci Rep. 2019 Feb 4;9:1175. doi: 10.1038/s41598-018-37602-7 (PMC6362254; doi:10.1038/s41598-018-37602-7)
Supplement: Supplementary file 1 — Supplementary material [file 41598_2018_37602_MOESM1_ESM.pdf]

Original association of ion transporters mediates the ECM-induced breast cancer cell survival: Kv10.1-Orai1-SPCA2 partnership.

Marta Peretti[1§], Mehdi Badaoui[1§], Alban Girault[1], Laurence Van Gulick[2], Marie-Pierre Mabille[1,3], Riad Tebbakha[1,3], Henri Sevestre[1,3], Hamid Morjani[2] and Halima Ouadid-Ahidouch[1*]

AUTHOR'S AFFILIATION:

[1]Laboratory of Cellular and Molecular Physiology, EA4667, University of Picardie Jules Verne, Amiens, France

[2]BioSpecT EA7506, Faculty of Pharmacy, Reims University, Reims, France

[3]Service d'anatomie pathologique and Tumor-library of Picardie, CHU d'Amiens, Université de Picardie Jules Verne, F-80000 Amiens, France

[§]Both authors contributed equally to this work

[*]Correspondence to: Halima OUADID-AHIDOUCH (ha-sciences@u-picardie.fr)

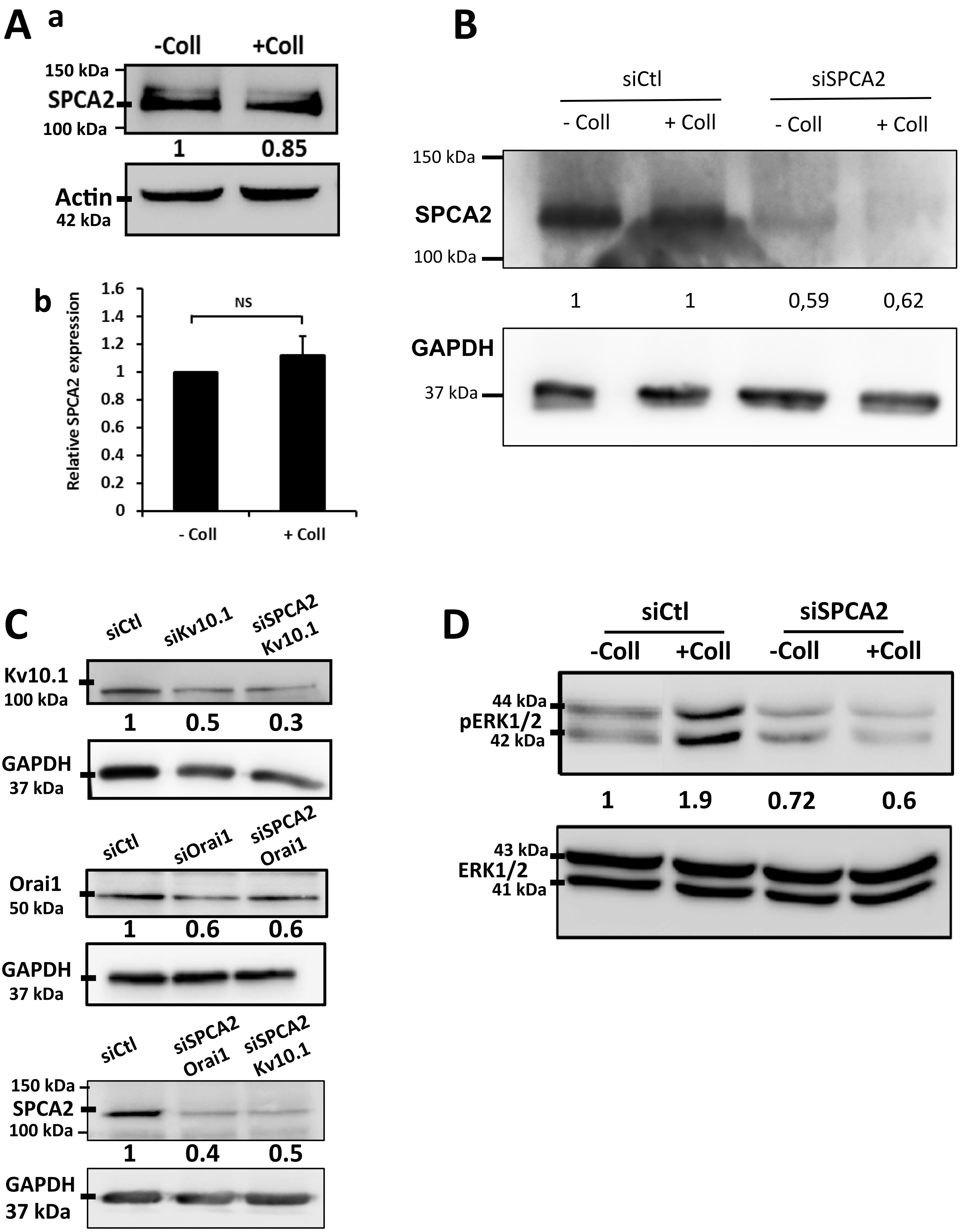

Suppl. Figure 1

Supp. Fig. 1- Effect of collagen 1 on SPCA2 expression and validation of siRNAs. Role for SPCA2 in ERK phosphorylation.

(A) Representative western blot (a) showing SPCA2 protein expression in MCF-7 cells after 48 h of starvation. Results were normalized as a percentage of not treated control condition. Values are reported as mean of all the experiments. Histograms (b) representing the averages ± standard error of results obtained by western blots. N=4, ns $p>0.05$, Two samples *t*-test **(B)** Representative western blot showing the efficiency of SPCA2 silencing in MCF-7 cells after 48 h of starvation. Results were normalized as a percentage of not treated control condition. Values are reported as mean of all the experiments. N=3 **(C)** Representative western blot showing the efficiency of siRNAs (siKv10.1, siOrai1 and siSPCA2) used in this work in MCF-7 cells after 48 h of starvation. Results were normalized as a percentage of control condition. Values are reported as mean of all the experiments. N=3 **(D)** Representative western blot showing ERK1/2 phosphorylation in siCtl and siSPCA2 MCF-7 cells after 48 h of starvation treated or not with collagen 1. Results were normalized as a percentage of not treated control condition. Values are reported as mean of all the experiments. N=3.

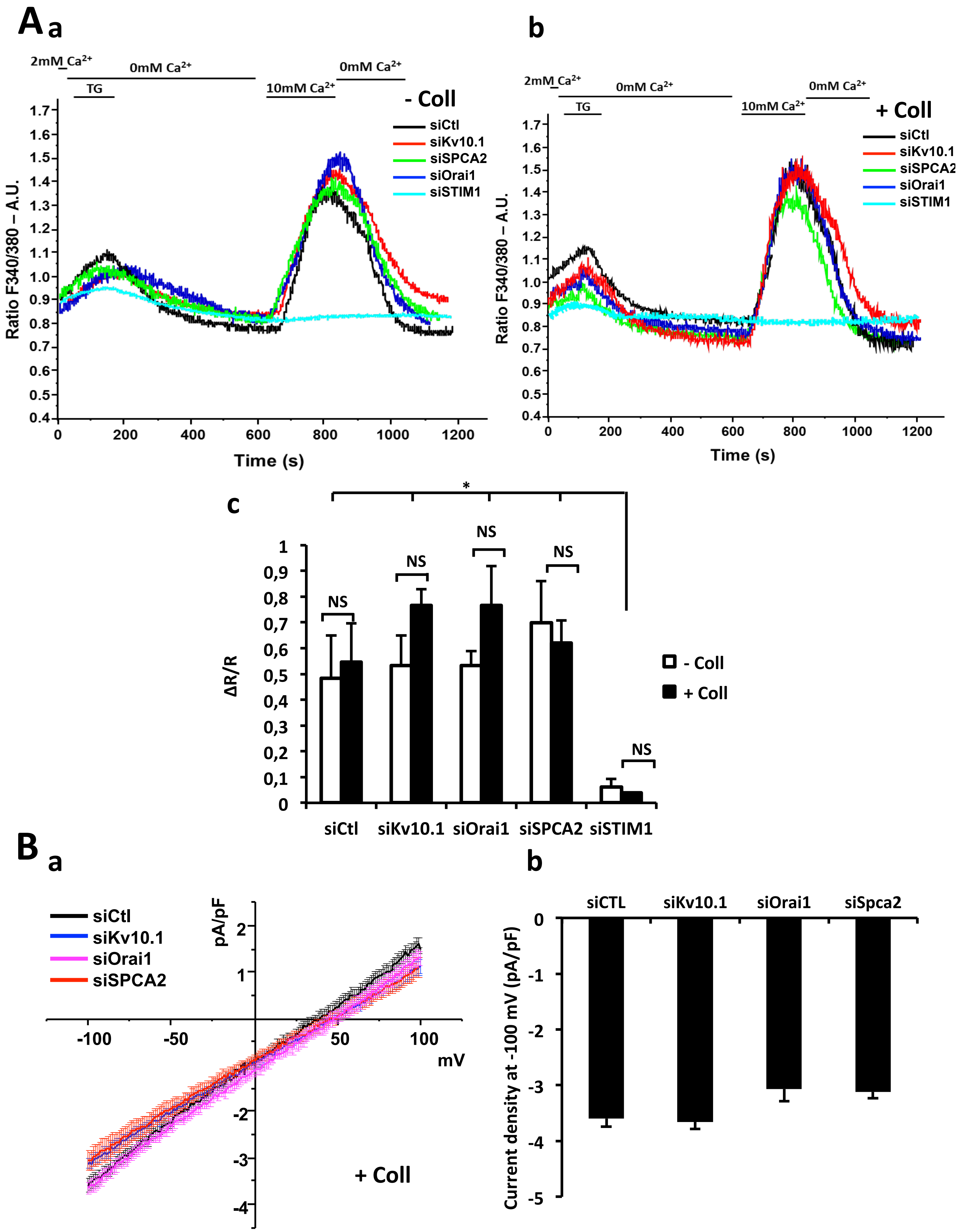

Suppl. Figure 2

Supp. Fig. 2- Collagen 1 does not induce a Store Operated Calcium Entry (SOCE).

(A) Representatives traces of SOCE in MCF-7 cells after 48 hours of starvation treated (b) or not treated (a) with collagen 1. (c) Histograms representing the averages ± standard error of SOCE: +Coll siCtl 0.55±0.15 (n=156), siKv10.1 0.77±0.06 (n=108), siOrai1 0.77±0.15 (n=120), siSPCA2 0.62±0.09 (n=104), siStim1 0.04±0.001 (n=101); -Coll siCtl 0.47±0.17 (n=146), siKv10.1 0.53±0.12 (n=112), siOrai1 0.53±0.06 (n=105), siSPCA2 0.7±0.16 (n=108), siStim1 0.06±0.03 (n=95) ; N=3 ; *$p \leq 0.05$, ANOVA followed by Holm-Sidak post hoc tests. (B) Patch clamp recordings of SOC currents. Whole cell currents (a) recorded in MCF-7 cells after 48 hours of starvation treated with collagen 1. The patch-clamp measurements were performed with specific bath solution to record SOC currents (see materials and methods section). 250 msec ramps from -100 mV to +100 mV from a holding potential of -40 mV were applied. Values are reported as mean ± standard error. (b) Histograms representing the averages ± standard error of currents values at -100 mV. Average current density values at -100 mV: siCtl -3.60±0.15 pA/pF, siOrai1 -3.07±0.22 pA/pF, siKv10.1 -3.12±0.11 pA/pF, siSPCA2 -3.66±0.13 pA/pF. siCtl and siSPCA2 (n=5), siOrai1 and siKv10.1 (n=4); no significant differences recorded; ANOVA followed by Tukey post hoc tests.

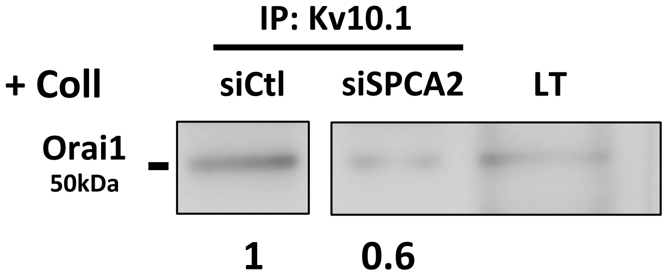

Suppl. Figure 3

Supp. Fig.3-Effect of SPCA2 silencing on Kv10.1-Orai1 interaction.

Representative western blot of Orai1 expression after immunoprecipitation with an anti-Kv10.1 antibody. Results were normalized as a percentage of the control condition. Values are reported as mean of triplicate experiments. Values are reported as mean of all the experiments. N=3.

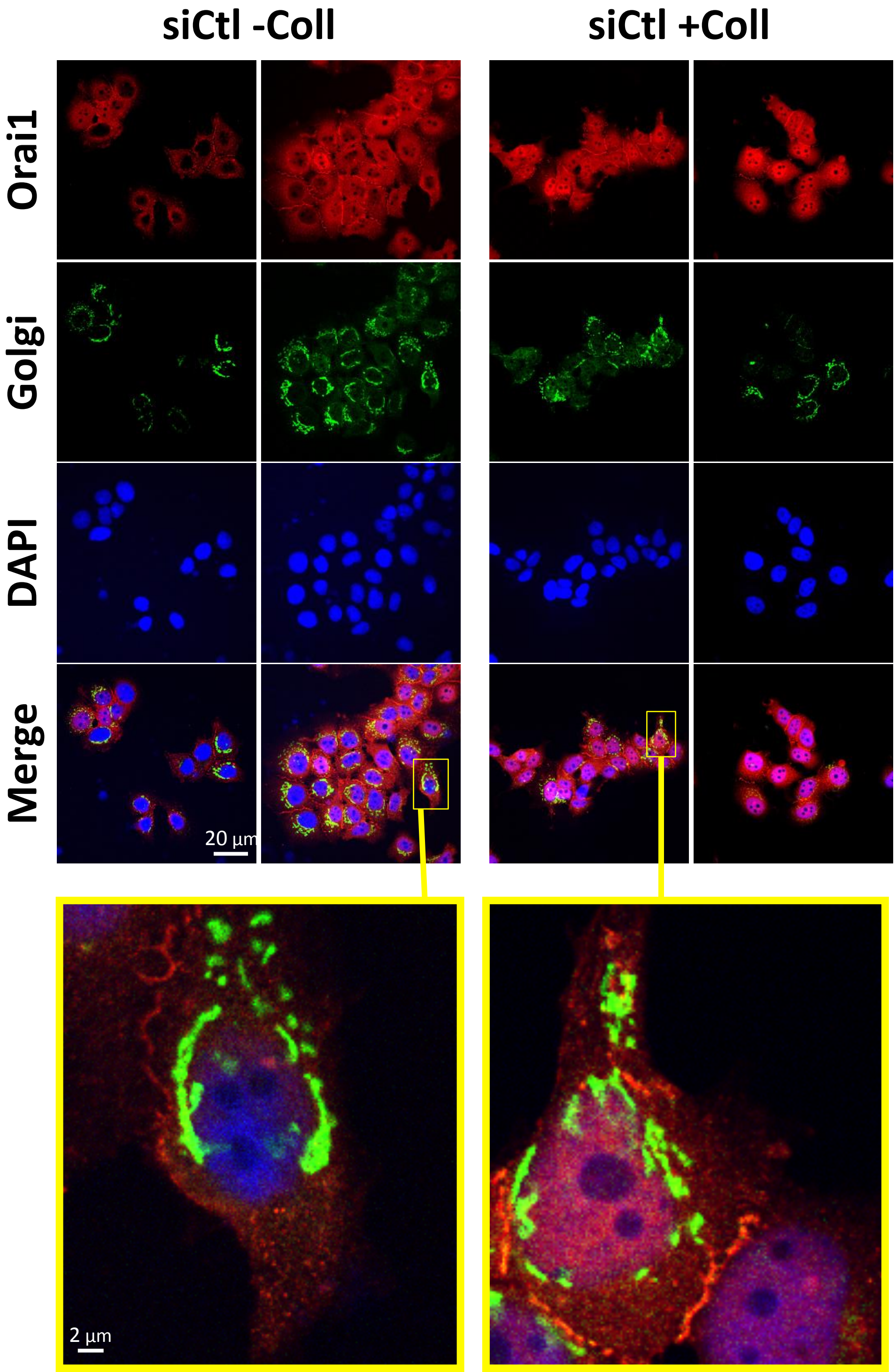

Suppl. Figure 4

Supp. Fig.4- Orai1 does not localize at a Golgi level

Confocal microscope images of MCF-7 cells infected with cell light Golgi-GFP vector at the beginning of the starvation and then immunostained with an anti-Orai1 antibody. The conditions analyzed were siCtl treated or not with collagen 1.

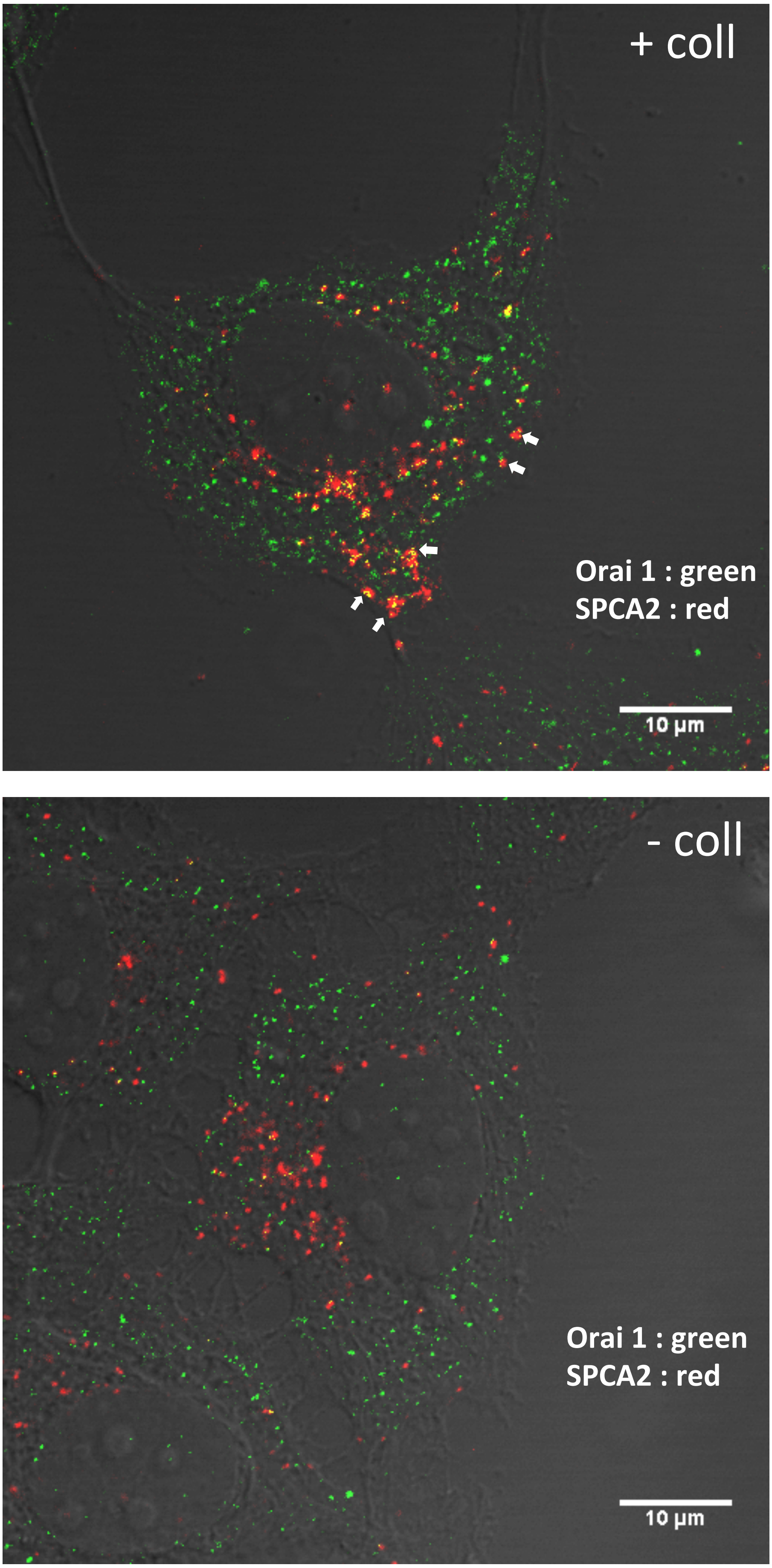

Suppl. Figure 5

Supp. Fig.5- High magnification of Orai1 and SPCA2 co-localization in MCF-7 cells seeded on collagen 1.

Confocal images of MCF-7 cells stained with anti-Orai1 and anti-SPCA2 antibodies seeded or not on collagen 1. High magnification of cells presented in figure 4. Co-localization of the two partners is significantly increased on collagen 1 matrix. The co-localization is at the plasma membrane level or closed to it.

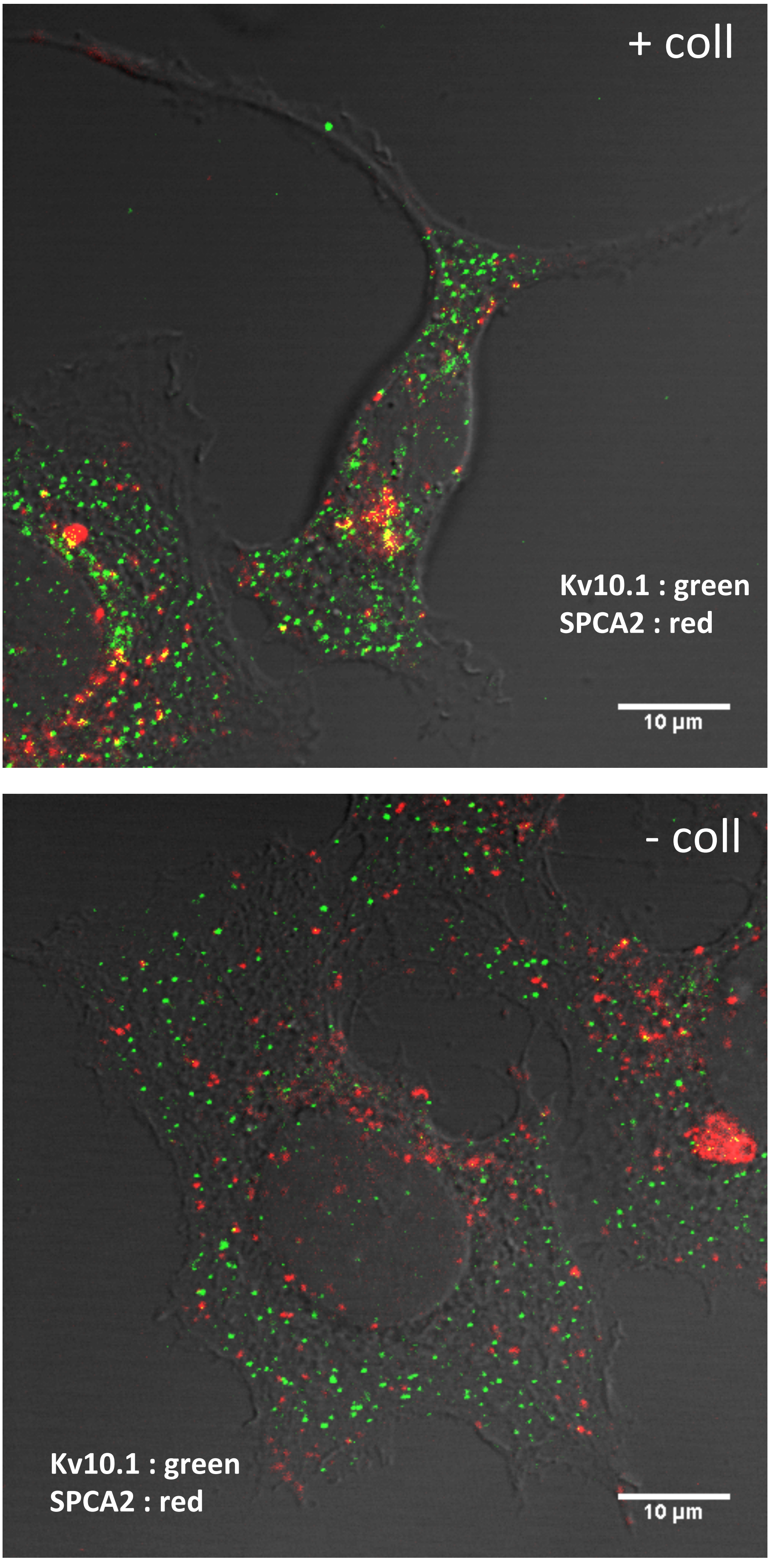

Suppl. Figure 6

Supp. Fig.6- High magnification of Kv10.1 and SPCA2 co-localization in MCF-7 cells seeded on collagen 1.

Confocal images of MCF-7 cells stained with anti-Kv10.1 and anti-SPCA2 antibodies seeded or not on collagen 1. High magnification of cells presented in figure 4. Co-localization of the two partners is significantly increased on collagen 1 matrix. The co-localization is preferentially at the intracellular level.

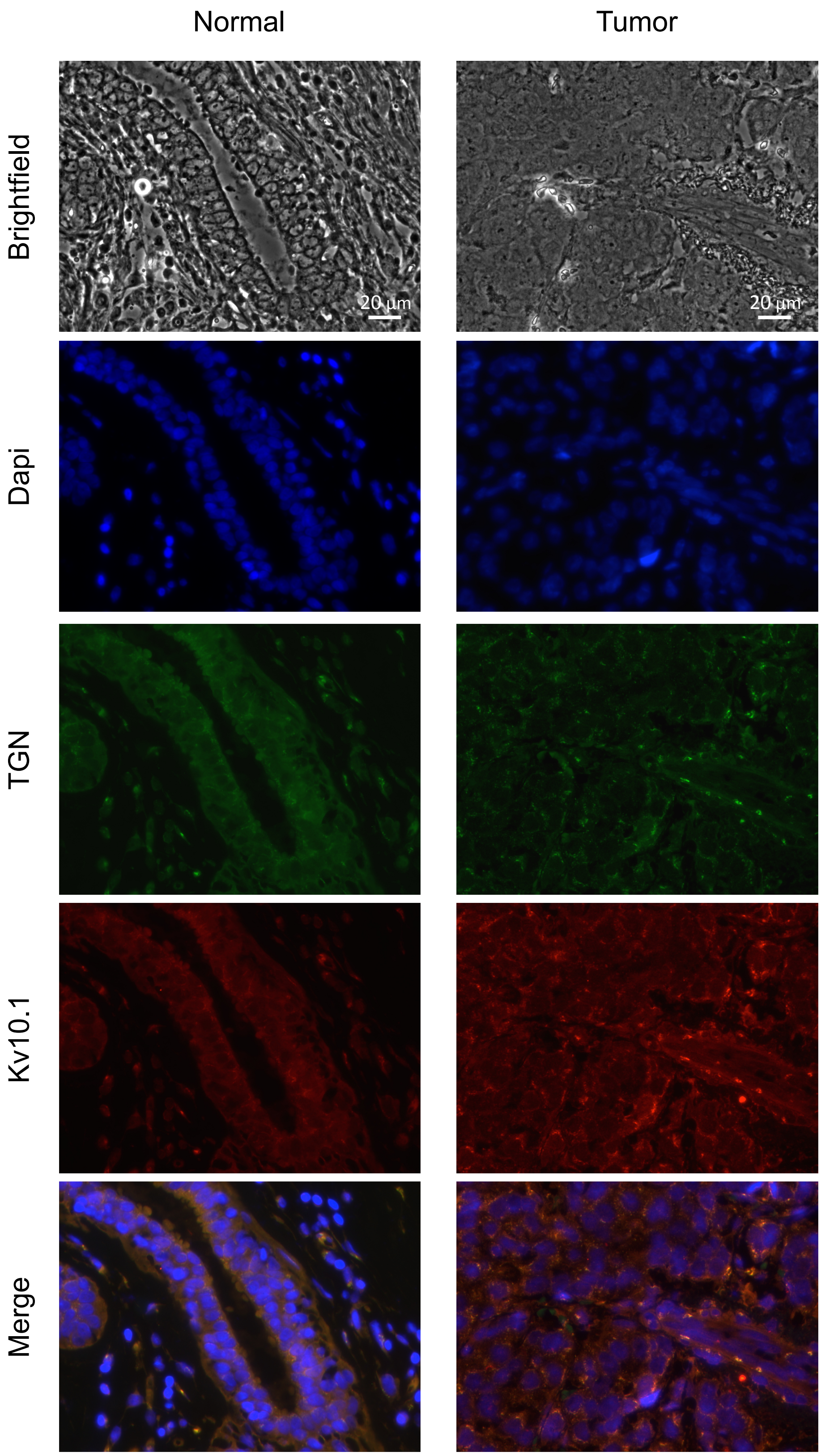

Suppl. Figure 7

Supp. Fig.7- Immunofluorescence staining of Kv10.1 in normal and tumor breast human tissue.

Representative immunofluorescent stainings of Kv10.1, and TGN-46 (TGN) in sections of normal and cancerous breast tissue. 40x magnifications of tumor (right column) is compared to normal (left column) tissues. In cancerous tissue, less colocalization of Kv10.1 with trans Golgi network is detected compared to the normal tissue.

Full length blots presented in the figures 4, 5 and 6

Figure 4 panel A

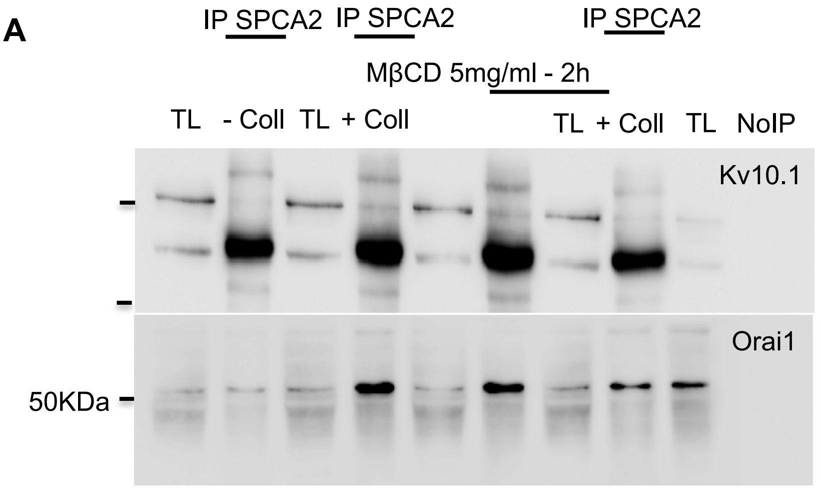

Figure 5 panel Aa

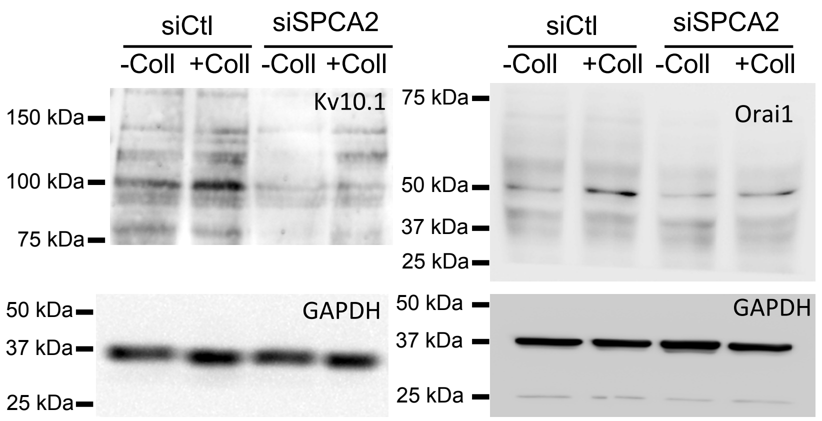

Figure 5 panel Ba and Bb

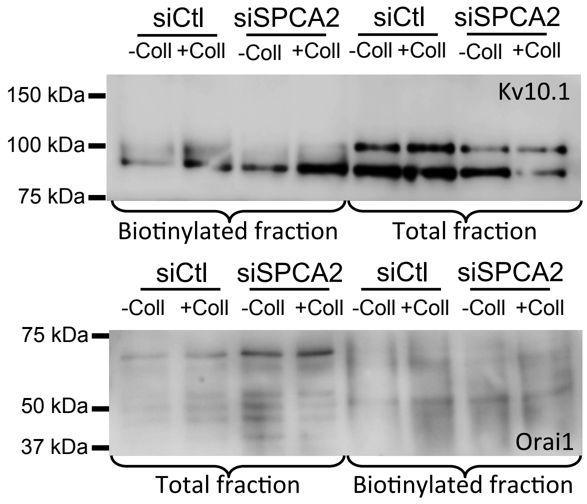

Figure 6 panel Aa

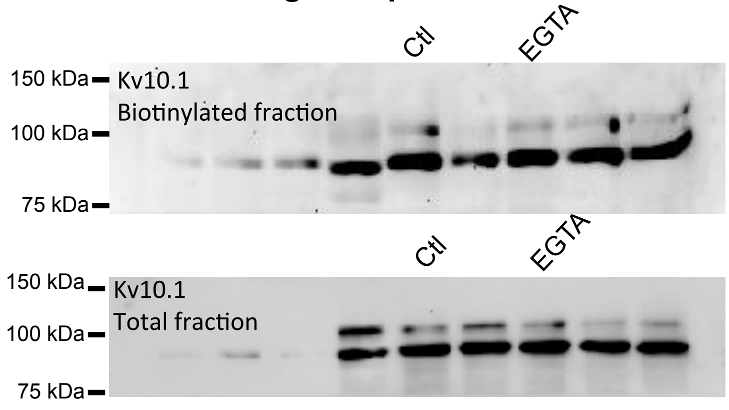

Figure 6 panel Ab

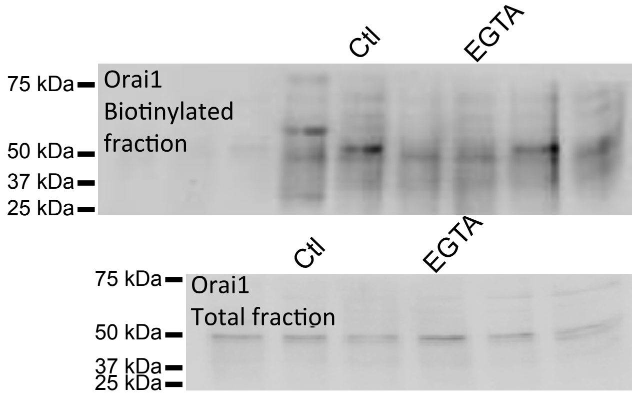

Figure 6 panel B

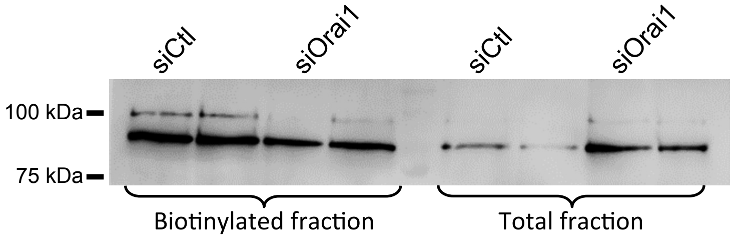

Suppl. Figure 8

Supp. Fig. 8- Full length blots used to compose the main body figures' article

Uncropped membranes of the western blot used in the article.

Full length blots presented in the supplementary material

Suppl. Figure 1 panel Aa

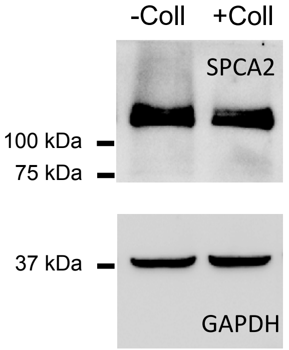

Suppl. Figure 1 panel B

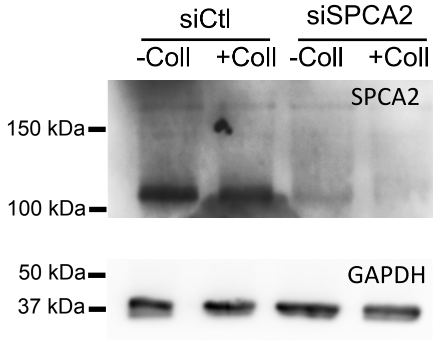

Suppl. Figure 1 panel C

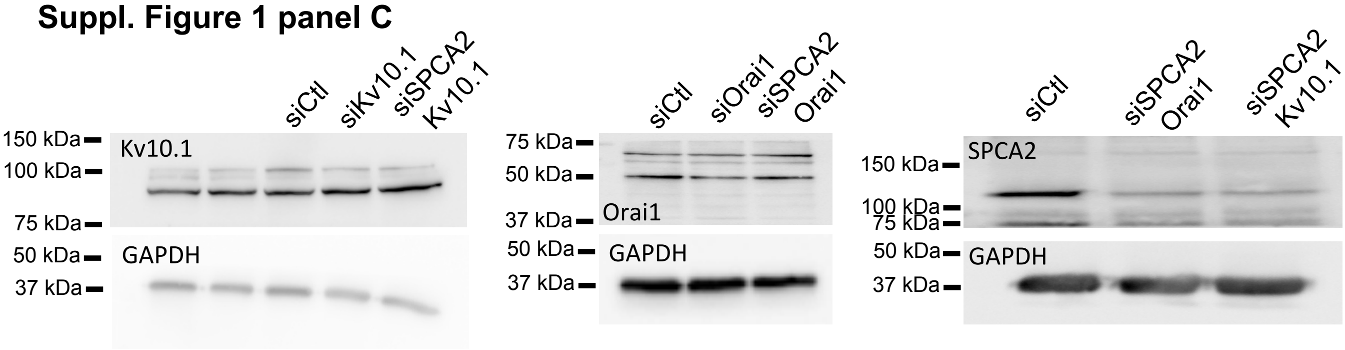

Suppl. Figure 1 panel D

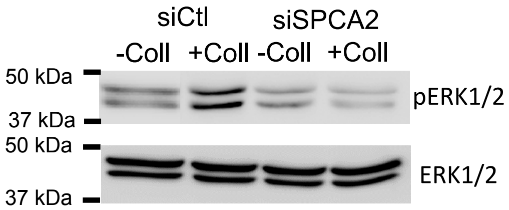

Suppl. Figure 3

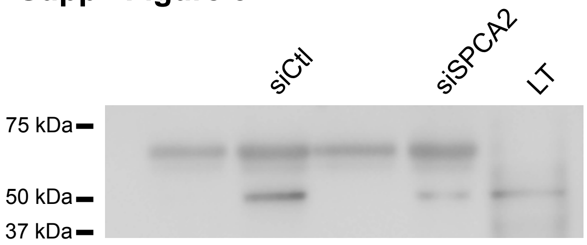

Suppl. Figure 9

Supp. Fig. 9- Full length blots used to compose the figure in the supplementary material section

Uncropped membranes of the western blot used in the supplementary material.
